# Anorganic Bovine Bone vs. Biphasic Calcium Phosphate in a Large Series of Maxillary Sinus Floor Elevations—A Non-Randomized Clinico-Morphological Study

**DOI:** 10.3390/jcm14238464

**Published:** 2025-11-28

**Authors:** Antonio J. Flichy-Fernández, Miguel Padial-Molina, Natividad Martin-Morales, Teresa Alegre-Domingo, Miguel Peñarrocha-Diago, Francisco O’Valle, Pablo Galindo-Moreno

**Affiliations:** 1 Department of Oral Surgery and Implantology, Valencia University Medical and Dental School, 46010 Valencia, Spain; m.teresa.alegre@uv.es (T.A.-D.); penarrochamiguel@gmail.com (M.P.-D.); 2 Department of Oral Surgery and Implant Dentistry, School of Dentistry, University of Granada, 18071 Granada, Spain; nati@ugr.es (N.M.-M.); pgalindo@ugr.es (P.G.-M.); 3 Instituto de Investigación Biosanitaria ibs.GRANADA, 18012 Granada, Spain; fovalle@ugr.es; 4 Department of Pathology, School of Medicine, University of Granada, 18071 Granada, Spain; 5 Institute of Biopathology and Regenerative Medicine (IBIMER), University of Granada, 18071 Granada, Spain

**Keywords:** sinus floor elevation, xenograft, alloplastic material, clinical study

## Abstract

**Background**: To compare the histological and histomorphometrical outcomes after sinus floor elevation using an anorganic bovine bone biomaterial or a biphasic calcium phosphate biomaterial. **Material and Methods**: Patients who needed maxillary sinus elevation were included in this study. A total of 68 implant sites were evaluated from a total of 42 patients. Twenty patients were treated with anorganic bovine bone, while 22 were treated with biphasic calcium phosphate biomaterial. Morphological and morphometrical studies were performed on the bone samples collected during implant placement. **Results**: Both biomaterials induced similar relative areas of mineralized tissue overall, particularly if only the area of grafted bone was considered. In turn, a higher proportion of non-mineralized tissue was observed in cases of biphasic calcium phosphate biomaterial with less area of remnant biomaterial particles. None of the implants failed at one year of follow-up. **Conclusions**: Although both biomaterials induce a similar amount of bone formation, the histopathological characteristics of the grafts are different, with a greater proportion of scar connective tissue with the biphasic calcium phosphate biomaterial.

## 1. Introduction

Bone regeneration is a technique that is used on a daily basis in many clinical practices. Such procedures are performed to complement the prosthetic rehabilitation techniques of many patients. These procedures aim to create an environment wherein the formation and maturation of a new bone substrate can occur, or to provide the necessary time for the biological process to be completed efficiently. In order to achieve the first objective, bone biomaterials are typically applied. Bone biomaterials are classified in four main categories according to their origin: autografts, allografts, xenografts, and alloplastic materials. The first three are derived from natural sources, while the fourth is a synthetic material. Although all these biomaterials demonstrate similar regenerative capacity [1], natural bone biomaterials are the most prevalent and well-understood. However, there is a growing inclination towards the utilization of synthetic biomaterials, among others, because of social principles of ecology and sustainability.

Among all biomaterials, the use of bovine xenograft is the most widely documented, both clinically and histologically, and serves as a standard of comparison with other biomaterials. It is true that biomaterials from different families may show significant differences in terms of histological results, but; nevertheless, most biomaterials from the same family and similar origin tend to show comparable characteristics in terms of composition, properties, and histological results [2], even if they undergo different industrial production processes [3].

The maxillary sinus model represents an optimal model for bone research in humans from an ethical aspect. It permits the observation of biomaterial maturation without the influence of external factors after performing relatively simple surgical techniques. This is achieved by taking samples at the time of implant placement, ensuring the integrity of the model. This study aims to examine the early healing and maturation of a biphasic calcium phosphate (BCP: 60% HA/40% beta-TCP) graft (EasyGraft Crystal+^®^), specifically designed for guided bone regeneration techniques such as sinus floor elevation, socket preservation, bone augmentation, or periodontal defects and peri-implantitis. Outcomes will be compared with those of an anorganic bovine bone (ABB) biomaterial (Cerabone^®^), which is used as a standard of comparison, in a large cohort of patients requiring sinus elevation.

## 2. Materials and Methods

### 2.1. Study Population

This study was designed as a parallel non-randomized cohort study based on the STROBE guidelines. The population under investigation was selected from subjects exhibiting a requirement for implant placement in the maxillary posterior sectors, with a residual bone height of less than 5 mm. The patients were treated at different private clinics in Valencia, Spain. All underwent sinus floor elevation surgery through the lateral window technique performed by the same operator (A.F.). The study protocol was approved by the Ethics Committee for Research in Humans of the University of Valencia (H1542027603961). All procedures were conducted in accordance with the Declaration of Helsinki’s principles. Prior to the commencement of any study-related procedure, written informed consent was obtained from all patients.

The inclusion was determined based on the patient’s age, which ranged between 25 and 70 years old (mean 45.8 (6.8)), and the quantity of remnant alveolar bone (less than 5 mm) according to the established criteria [4], and the inclusion of only physically and mentally healthy patients.

The exclusion criteria were as follows: patients with acute or chronic sinusitis, any type of paranasal sinus pathology detectable on CBCT, individuals with pre-existing medical conditions (e.g., uncontrolled diabetes, heart disease, etc.), diseases or medication intake that could alter bone metabolism, pregnancy and lactation, uncontrolled periodontal disease, and inadequate dental hygiene habits.

### 2.2. Surgical Protocol

The patients were anesthetized with local infiltration of an anesthetic compound with articaine. A crestal incision was then made with a mesial vertical incision, and a full-thickness flap was raised. A lateral bone window was created 1–2 mm from the base of the maxillary sinus with a handpiece and a number 8 tungsten carbide ball bur. The Schneiderian membrane was separated and elevated with sinus tools, separating the mesial, distal, occlusal, and palatal areas. The vestibular wall of the bone window was kept attached to the Schneiderian membrane and placed perpendicularly, thus forming the roof of the area to be grafted. The cavity was filled with the biomaterial indicated in each case (Biphasic Calcium Phosphate, BCP, EasyGraft Crystal+^®^, Collagen Matrix, Inc., Oakland, NJ, USA, or Anorganic Bovine Bone, ABB, Cerabone^®^, Botiss Dental, Berlin, Germany), from the distal and palatal, and mesial and palatal, towards the middle and anterior areas to completely fill the elevated area. Finally, a pericardial membrane was placed to cover the lateral window (Jason^®^ Membrane, Botiss Dental, Germany). It was sutured with simple 4/0 vycril stitches. In all cases, Amoxicillin 750 mg every 8 h for 7 days, Ibuprofen 600 mg every 8 h for 5 days, and 0.12% chlorhexidine mouthwash were prescribed. The suture was removed after 7 days, and clinical controls were carried out every month to observe the evolution.

After 6 months, the control CT was performed before the placement of the dental implants to check that an adequate height gain had been achieved. In the second surgical procedure, 6 months after the sinus lift, the patient was anesthetized with an infiltration technique with articaine. The incision was made with a punch, and a trephine was used to take a bone sample, and then the drilling sequence was continued. Finally, the dental implants were placed with a closing cap. The same medication was prescribed as in the sinus floor elevation surgery. Three months after the placement of the dental implants, the second phase was carried out to place the healing abutments and subsequently perform the prosthetic restoration of the area, with a final six-monthly radiological checks.

### 2.3. Data Recorded

Clinical data recorded included age, gender, and complications of intra- or postsurgical procedures. Additionally, CBCTs were performed before the sinus floor elevation surgery and 6 months later, immediately before implant placement. This allowed us to calculate the radiographic crestal bone height before the procedure, which was useful for the placement of implants.

### 2.4. Histopathological Analysis

After a six-month healing period of the sinus lift technique, during the implant placement surgeries, bone samples were obtained from these grafts for histological and histomorphometric analysis. The surgeon who performed the sinus elevations was also in charge of placing the implants and collecting the samples. Tissue samples were sequentially coded so that the type of graft used in each particular case was not known to the pathologist. The grafted areas were penetrated with 4 mm external diameter trephines, and the samples obtained were immediately fixed in 10% buffered formalin for 48 h at room temperature. Then, they were transferred to 70% ethanol and subsequently sent to the Biomedical Research Center of the University of Granada (CIBM-UGR), where they were processed and evaluated. Upon arrival, the samples were subjected to decalcification for a period of 48 h at 37 °C, utilizing the Decalcifier I solution (Surgipath Europe Ltd., Peterborough, UK). Thereafter, the samples were embedded in paraffin. The specimens were processed by obtaining Sections (4 μm) taken along the corono-apical axis of each specimen. Subsequently, the samples were deparaffinized, rehydrated, and stained using conventional hematoxylin and eosin, and Masson’s trichrome techniques. The number of osteoblasts, osteoclasts, and osteocytes per mm^2^ was quantified using a scale-coupled microscope (BH2, Olympus Optical Company Ltd., Tokyo, Japan). The histomorphometric quantification was conducted semi-automatically with Masson’s trichrome-stained sections, encompassing the analysis of the fractions of mineralized tissue, non-mineralized tissue, and biomaterial remnant tissue. For the purpose of cell histological analysis, ten random images of each sample were captured with a X10 objective using a microscope equipped with a digital camera (DP70, Olympus Optical Company Ltd.). The evaluation of the tissue compartments described above was conducted using the ImageJ 1.54 software (NIH, https://imagej.net/ij/). All histological evaluations were performed by an experienced pathologist who was unaware of the type of biomaterial used (F.O.).

### 2.5. Statistical Analysis

For each type of variable, percentages, means, and standard deviations were calculated, depending on whether the data were categorical or continuous. Categorical data were evaluated with the chi-square test while continuous data were analyzed using the Mann–Whitney U Test in IBM^®^ SPSS^®^ 28.0 for MacOS software (IBM Corporation, Armonk, NY, USA). Statistical significance was set at *p*-values < 0.05.

## 3. Results

This section may be divided by subheadings. It should provide a concise and precise description of the experimental results, their interpretation, and the experimental conclusions that can be drawn.

### 3.1. Clinical Variables

As shown in Table 1, a total of 68 implant sites were evaluated, thus collecting 68 samples, from a total of 42 patients. Twenty patients were treated with ABB, providing 30 samples, while 22 were treated with BCP, providing a total of 38 samples.

Regarding bone height before and after the surgical procedure, both biomaterials provided a change from approximately 3 mm in height to around 9 mm. The differences were not statistically significant at any time point.

None of the implants failed at one year of follow-up.

### 3.2. Histopathological Results

**Figure 1:**
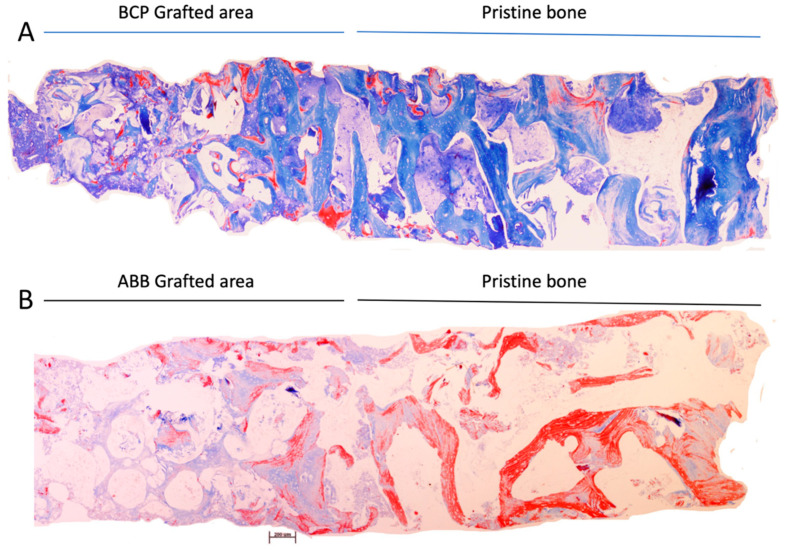
Panoramic microphotography of maxillary sinus lift biopsies. (**A**) BCP. (**B**) ABB. Note the formation of new bone surrounding the biomaterial in the graft zones. Masson’s trichrome staining. Scale bar: 200 μm.

**Figure 2:**
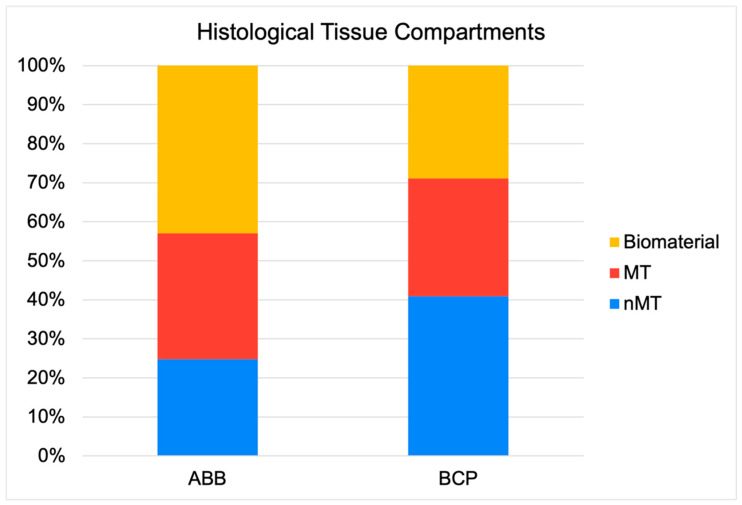
Graphical representation of the relative area occupied by different bone components in the BCP and ABB groups.

As shown in Table 2 and Figure 1, the cores obtained had similar length (5.26 (1.96) vs. 5.12 (2.05); *p* = 0.805) and area (6.71 (2.93) vs. 7.06 (3.25); *p* = 0.863; ABB vs. BCP, respectively; Mann–Whitney U test). Likewise, within the area of pristine bone (non-grafted remnant bone crest), both the mineralized tissue and non-mineralized tissues occupied a similar relative area. Both biomaterials induced similar relative areas of mineralized tissue in the grafted area (32.24 (13.71) vs. 30.19 (13.69); *p* = 0.727; ABB vs. BCP, respectively; Mann–Whitney U test). As also shown in Table 2, higher proportion of non-mineralized tissue was observed in cases of BCP (24.82 (13.31) vs. 40.90 (19.69); *p* = 0.002; ABB vs. BCP, respectively; Mann–Whitney U test) with less area of remnant biomaterial particles (42.94 (14.88) vs. 28.21 (19.42); *p* = 0.002; ABB vs. BCP, respectively; Mann–Whitney U test) (Figure 2), which demonstrates a higher maintenance of ABB in the tissue because of less resorption.

**Figure 3:**
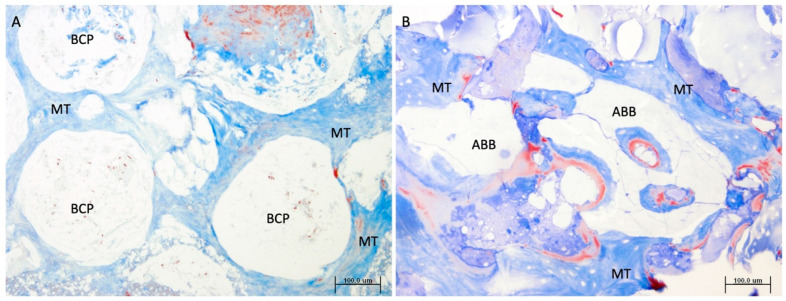
Microphotography of maxillary sinus lift biopsies. (**A**) BCP. (**B**) ABB. Note the formation of new mineralized tissue (MT) surrounding the biomaterials (BCP, ABB) and inside the pores in the case of ABB in the grafted areas (Masson’s trichrome staining, original magnification 10×). Scale bar: 100 μm.

Although more osteoblasts were observed in the grafted area of the BCP group compared to ABB (64.52 (75.77) vs. 125.24 (86.54); *p* = 0.002; ABB vs. BCP, respectively; Mann–Whitney U test), the remodeling activity considering the number of osteoid lines was similar in both groups, although, as expected, it was higher in the grafted area (2.52 (2.67) vs. 2.93 (2.25); *p* = 0.326; ABB vs. BCP, respectively; Mann–Whitney U test) than in pristine bone (0.80 (1.16) vs. 1.20 (1.28); *p* = 0.160; ABB vs. BCP, respectively; Mann–Whitney U test). The greater microporosity of BCP determined the presence of cells inside the biomaterial. Thus, regarding cellularity, more osteocytes were observed in the BCP (284.95 (226.85) vs. 539.85 (195.54); *p* < 0.001; ABB vs. BCP, respectively; Mann–Whitney U test). The number of osteoclasts was similar in both groups (Figure 3).

On the ABB particles, the formation of bone spicules was identified inside the pores in addition to the periphery of the particles in greater proportion. In the BCP particles, due to their physical-chemical characteristics, proliferation of fusiform cells of different nature inside and around them was observed, but less new bone was formed around particles (Figure 3).

## 4. Discussion

The aim of this work was to evaluate the differences in terms of healing and maturation of different nature bone grafts after 6 months of healing in a human model of sinus lift. Although the differences were not notable in terms of new mineralized component, the 40% beta tricalcium-phosphate (beta-TCP) and 60% hydroxyapatite (HA) biomaterial showed a higher resorption rate and a higher osteogenic cellular component, although no more osteogenic formation lines, than the sintered anorganic bovine bone biomaterial.

In the present study, two commercially available biomaterials were used. Cerabone^®^ was used as a reference material, as it belongs to the bovine xenograft family. One of the representatives of this family, Bio-Oss^®^, is the most documented biomaterial, but in comparative studies with Cerabone^®,^ both biomaterials show very similar biological and structural properties [5]. Nevertheless, we elected to use Cerabone^®^ given that histological outcomes for this biomaterial are scarce in the literature; thus, through this study, we could contribute data to the scientific community. Conversely, the comparison was conducted with a synthetic biomaterial, EasyGraft Crystal+^®^, a biphasic calcium phosphate (60% HA/40% beta-TCP), indicated for sinus lift techniques, among others.

Cerabone^®^ (Botiss Dental, Berlin, Germany) is a sintered anorganic bovine bone biomaterial, with particles about 0.5 to 1 mm, with a very high hydrophilicity, pure mineral ~100% hydroxyapatite composition, high crystallinity, and an insignificant level of impurities ([6]. The calcium release from this biomaterial by dissolution in liquid is low, but osteoclast-induced calcium and phosphate release is intermediate in comparison with other biomaterials ([7]), what could promote a slower resorption rate? These findings may justify the observation that grafts composed of this biomaterial exhibit less volumetric reduction than those composed of other anorganic bovine bones [5].

EasyGraft Crystal+^®^ (Collagen Matrix, Inc.) is 40% beta tricalcium-phosphate (beta-TCP) and 60% hydroxyapatite (HA), with a granule size of 0.45 to 1 mm, coated by a 10 µm layer of Poly lactic-co-glycolic acid (PLGA), in order to improve its handling and adhesive properties ([8]). The PLGA addition to this type of particle has been observed to enhance vascularization of the graft and to increase the number of osteoprogenitor cells, despite the absence of radiological, histological, or histomorphometric advantages over the standard biomaterial ([9]).

Comparing our results with other studies performed with these same biomaterials, we found that the results are reproducible with those previously reported. With regard to the use of Easy Graft Crystal+^®^, previous studies found histomorphometric values very similar to the present study. Thus, in a comparative study published by our group in 2019, the histomorphometric results for this biomaterial were very similar to those from the current study: 31.25 (13.82) % of new mineralized tissue, compared to 30.19 (13.69) % in the present study; 46.0 (16.63) % of non-mineralized component versus 40.90 (19.69) %, in the current study; and 23.38 (24.52) % of remaining biomaterial in comparison to 28.91 (19.42) % in this study [9]).

However, our histomorphometric results obtained from the ABB grafts were somewhat different from those reported by other authors, especially with regard to the duality of non-mineralized tissue/remaining biomaterial. In our study, we found 32.24 (13.71) % of new mineralized bone structure, 24.82 (13.31) % of non-mineralized bone component, and 42.94 (14.88) % of remnant biomaterial. However, Tawil and coworkers reported 26.15 (11.18) % of mineral bone, 46.03 (5.84) % non-mineralized tissue, and 27.82 (11.97) % remaining biomaterial [10]). Similar results were reported by Pereira and collaborators with 25.94 (10.55) % mineral structure, 51.00 (10.31) % non-mineral structure, and 20.34% remaining biomaterial ([11]). Klassmann and colleagues, also using the maxillary sinus model, showed completely discordant results, reporting 43.1 (5.2) % mineral bone structure, 26.0 (2.2) % non-mineral bone structure, and 22.5 (2.8) % ABB remnant ([12]).

Data derived from our research are interesting because, despite the fact that BCP has resorbed more, the number of osteoclasts is almost twice in the grafts composed of ABB, although without statistically significant differences. This greater presence of osteoclasts after 6 months of maturation in these grafts may be due precisely to the significantly greater long-term persistence of the bovine biomaterial or different mechanisms of dissolution in the case of phosphate biomaterials.

It has been widely discussed in the literature that the resorption of biomaterials is essentially due to two types of biological processes: 1.—The physico-chemical degradation of the biomaterial in the biological environment where it is placed [7], and 2.—The direct osteoclastic action on the particles, which can be evaluated both by the presence of osteoclast cells and/or by the proteins that these cells secrete to exert their biological action, either tartrate-resistant acid phosphatase (TRAP), cathepsin k, or osteopontin ([13]). Synergistically, a biomaterial that possesses a high degree of osteoconduction, as in the case with anorganic bovine bone, is necessarily a biomaterial with a low resorption rate, as both osteoclasts and fluids have great difficulty accessing the core of the biomaterial once it has been completely surrounded by a new mineralized bone matrix. In these cases, it is consistent to find the osteoclasts specifically located on the part of the particle that is not surrounded by new bone.

Our results may explain the possibility that both biomaterials follow different resorption processes and different degradation rates. It is evident that the 40% beta-tricalcium-phosphate (beta-TCP) and 60% hydroxyapatite (HA) biomaterial degraded quickly than anorganic bovine bone, which in turn resulted in a significant increase in the non-mineralized bone component in BCP grafted sinuses. In fact, the early disappearance of the biomaterial has been advocated as one of the ideal properties of biomaterials and is considered to be one of the biological advantages of ß-tricalcium phosphate, as it tends to be rapidly resorbed [14].

It is obvious that the final composition of the new bone is formed by the partial amount of its components. In the case of bone grafts based on the use of non-autogenous biomaterials, this final composition is the sum of the percentages of the new mineralized bone component, the non-mineralized bone component, and the biomaterial particles that persist. Well, if we analyze these three components in our new bone, it is easy to appreciate that:

1. Both biomaterials have the same capacity to form mineral bone structure. This is to be expected, since bone formation depends on the genetics and function of the new bone to be formed, very much conditioned by the location where the graft is placed, and not on the action of the biomaterial itself, since this is usually only a scaffold, necessary for cell apposition and the promotion of osteoconduction and later osteogenesis. Avila–Ortiz et al. reported that no biomaterial or combination of biomaterials is able to overcome the characteristics and quantity of pristine bone typical of the grafted area [1]. In this sense, it is important not only to consider that the percentage of new mineralized bone tissue formed by both biomaterials is similar, but also that the osteogenic activity, assessed by the number of new osteoid lines, in both grafts (2.52 (2.67) vs. 2.93 (2.25); *p* = 0.326; ABB vs. BCP, respectively; Mann–Whitney U test).
2. Regarding the presence of remnant biomaterial, BCP disappears earlier in the newly formed bone, perhaps due to a different resorption mechanism than ABB or a different response to the osteoconduction mechanism. From the histological observation of these samples, it can be clearly seen how the pattern of formation of new mineral structure appears different between the two types of biomaterial particles. While the anorganic bovine bone particles seem to establish an intimate contact with the new bone mineral structure, the beta tricalcium-phosphate (beta-TCP) granules seem to allow bone formation in a more eccentric way, maintaining the porosity between the granules in a more intact way, which could facilitate the earlier physico-chemical action of the fluids and proteins of the tissue microenvironment. On the other hand, comparatively, anorganic bovine bone is much more crystalline in nature and somewhat more rigid in structure, which may slow the rate of bone resorption [15]. As mentioned above, the manufacturing process of each biomaterial can change its morphological, structural, or chemical properties, even if they are of the same origin, which may change the clinical results [16]. For example, Trajkovski et al. determined that the sintering process performed on Cerabone^®^ leads to an increase in crystalline size, which would provide better long-term volume stability of this biomaterial compared to low-temperature or chemically treated bovine biomaterials [6].
3. The sum of the new mineral bone structure and the remaining biomaterial obviously determines the amount of non-mineralized vital bone, also known in the literature as ‘soft connective tissue’. Therefore, in this comparative study, the same as the new bone mineral structure, the amount of remaining biomaterial inversely conditions the amount of non-mineralized bone tissue, this component being greater in the graft composed of Easy Graft+^®^ than in the graft composed of ABB. The function of this tissue component is depreciated in Implant Dentistry, since grafts with more non-mineralized structure are attributed with a lesser primary stability or insertion torque of implants, suggesting lower clinical mechanical retention. However, it is worth noting that it is in this component where stem cells and micro-vascularity accumulate and enhance the bone healing around implants and homeostasis phenomena of this resultant bone. Consequently, due to a greater quantity of non-mineralized bone component in the grafts made with Easy Graft+®, it is also logical to observe in them a greater osteoblastic cellularity, measured in a greater significant number of osteoblasts, and finally a greater osteocyte density, as has occurred in our comparison. The presence of polylactic-co-glycolic acid (PLGA) on the surface of Easy Graft+^®^ particles may also contribute to the increased cellularity found in comparison to grafts made with anorganic bovine bone. In a previous study, we demonstrated that the presence of PLGA, an osteoconductor that facilitates the adhesion of preosteoblasts and osteoblasts to the matrix surface [17], also promoted an increase in the number of mesenchymal stem cells and microvascular density in the non-mineralized tissue of grafts made of these biomaterials, compared to similar grafts without the presence of PLGA [9].

As previously demonstrated by Stacchi and colleagues, maxillary remnant bone does not appear to have played any role in the histomorphometric results obtained with both grafts [18]. In fact, there were no differences in the histomorphometric components of this bone in both comparison groups in terms of mineralized and non-mineralized tissue. Similarly, there was no difference in the osteogenic activity of both groups, represented by the presence of osteoid lines. Although Rios et al. demonstrated that the height of the remnant bone played no role in the final graft outcome [19], in our study, there was no initial difference between the two experimental groups regarding this variable.

Our study has a main limitation, as it is not a randomized study. It would have been ideal to establish a split-mouth model to compare the healing of both biomaterials in the same subjects. However, nowadays, it is complicated to recruit patients with both sides of the maxilla in need of sinus augmentation. In addition, randomization for the current study was also not possible as other conditioning factors intervened in the biomaterial selection, particularly the clinical setting, so that some practices advocated the use of animal-derived biomaterial while others preferred to provide their patients with synthetic ones. Also, the statistical power achieved in the current study with the sample size we have been able to select is low. But, in any case, the information obtained here can be used to define future randomized studies to confirm our findings. In addition, the time frame of the study, although ideal for the histological evaluation of the bone at the time of implant placement, is insufficient to evaluate long-term remodeling, volumetric stability, or the clinical performance of implants. In any case, implant performance was not an objective of this study, which, as stated, aimed at providing an evaluation of the initial bone healing and maturation depending on the use of two different biomaterials.

## 5. Conclusions

Although both biomaterials induce a similar amount of bone formation, the histopathological characteristics of the grafts are different, with a greater proportion of scar connective tissue when using a biphasic calcium phosphate (EasyGraft Crystal+^®^). The impact of such differences in clinical or radiographical variables remains to be studied.

## Figures and Tables

**Table 1:** Summary of clinical and radiographical variables and statistical comparison between groups. Unless otherwise noted, data is reported as mean (SD).

	ABB	BCP	*p* Value
Age (mean (min-max))	45 (36–62)	47 (38–63)	0.464
Gender (n (%))			0.142
Female	12 (60.0%)	8 (36.4%)	
Male	8 (40.0%)	14 (63.6%)	
Bone Height Before (mm)	2.99 (0.57)	2.93 (0.52)	0.700
Bone Height After (mm)	8.98 (0.77)	9.05 (1.01)	0.666

*p* value is indicated using the Mann–Whitney U Test except for Gender, where the Chi-square test was used.

**Table 2:** Summary of histological and histomorphometrical variables and statistical comparison between groups. Unless otherwise noted, data is reported as mean (SD).

	ABB	BCP	*p* Value ^1,2^
Length of the biopsy (mm)	5.26 (1.96)	5.12 (2.05)	0.805
Total area of the biopsy (mm^2^)	6.71 (2.93)	7.06 (3.25)	0.863
Relative area of mineralized tissue in pristine bone (%)	53.81 (17.09)	54.57 (15.83)	0.912
Relative area of non-mineralized tissue in pristine bone (%)	46.19 (17.09)	45.43 (15.83)	0.912
Relative area of mineralized tissue in grafted bone (%)	32.24 (13.71)	30.19 (13.69)	0.727
Relative area of non-mineralized tissue in grafted bone (%)	24.82 (13.31)	40.90 (19.69)	0.002
Relative area of remnant biomaterial in grafted bone (%)	42.94 (14.88)	28.91 (19.42)	0.002
Osteoid Lines in pristine bone (n)	0.80 (1.16)	1.20 (1.28)	0.160
Osteoid Lines in grafted bone (n)	2.52 (2.67)	2.93 (2.25)	0.326
Osteocytes in grafted bone (n/mm^2^)	284.95 (226.85)	539.85 (195.54)	<0.001
Osteoblasts in grafted bone (n/mm^2^)	64.52 (75.77)	125.24 (86.54)	0.002
Osteoclasts in grafted bone (n/mm^2^)	76.34 (120.25)	47.44 (71.13)	0.424

^1^ Italics indicated statistically significant value. ^2^ *p* value is indicated using the Mann–Whitney U Test.

## Data Availability

The data that support the findings of this study are available from the corresponding author upon reasonable request.
